# Brief Research Report: Effect of Triiodothyronine on Hepatic Growth Hormone Receptor Expression in Primary Bovine Hepatocytes

**DOI:** 10.3389/fvets.2022.882102

**Published:** 2022-06-17

**Authors:** Vera Stiensmeier, Marion Schmicke

**Affiliations:** Animal Health Management, Institute of Agricultural and Nutritional Sciences, Faculty of Natural Sciences III, Martin-Luther University Halle-Wittenberg, Halle, Germany

**Keywords:** hepatocytes, Cattle, growth hormone, growth hormone receptor, triiodothyronine

## Abstract

In previous studies, triiodothyronine (T3) was found to be lower in cows with ketosis and an effect of T3 on Growth Hormone Receptor (GHR) expression is described, e. g., in a human hepatoma cell line. Therefore, this study aimed to test whether T3 affects GHR messenger RNA (mRNA) expression in a well-established bovine hepatocyte model. Hepatocytes were kept in a sandwich culture and stimulated for 6 days with constant (10 μg/ml) or decreasing (from 10 to 5 μg/ml) T3 concentrations, and GHR, as well as IGF-1 mRNA expression, was measured using real-time polymerase chain reaction (RT-PCR). We could confirm *in vitro* that T3 has a stimulatory effect on GHR1A mRNA expression.

## Introduction

At the onset of lactation in dairy cows, the somatotropic axis becomes uncoupled due to a hepatic Growth Hormone (GH)-resistance by decreasing Growth Hormone Receptor (GHR) expression, resulting in an increase of GH and a decline of IGF-1 (1–3). This mediates a metabolic adaption as high GH levels stimulate lipolysis and gluconeogenesis, thereby supporting milk yield. Insufficient adaption can lead to metabolic disorders like ketosis. Also, within the thyrotropic axis changes occur, which lead to an adjustment of metabolism in the transition period (4). The onset of lactation results in the systemic decline of thyroxine (T4) and metabolically active triiodothyronine (T3) (5), but increasing concentrations of 5′deidonisase and with that T3 in the mammary gland (6, 7). That results in a metabolic prioritization of the mammary gland (6), but downregulation of metabolic demands in the periportal period (8). In ketotic cows, a decline of thyrotropic hormones, as well as hepatic GHR expression, was previously detected (9, 10). A direct stimulatory effect of T3 on GHR expression was previously demonstrated in, for example, human hepatoma cells (11). The effect of T3 is mediated by various intracellular pathways and directly at nuclear thyroid hormone receptors. This study aimed to test whether the active form of thyroid hormones (T3) may have an additive and direct effect on hepatic GHR mRNA expression *in vitro* in bovine primary hepatocytes.

## Methods

Hepatic tissue used in this study came from a donor animal (Deutsche Holstein, female, 2 years old, non-lactating) that was euthanized in the Clinic for Cattle, University of Veterinary Medicine, Hanover, Germany because of an unfavorable prognosis due to lead contamination. A clinical chemistry revealed that liver values were normal (bilirubin 5.5 μmol/l reference of <7; aspartate aminotransferase 59 U/l reference <100; gamma-glutamyl transferase 29 U/L reference <33) or slightly elevated (glutamate dehydrogenase 25.8 U/L reference <14). Primary bovine hepatocytes were isolated and cultivated according to an established protocol by Witte et al. (12), with the following slight modifications: For purification of hepatocytes, Percoll^®^ (REF P4937; Sigma-Aldrich Merck KGaA, Darmstadt, Germany) was applied. The viability of cells was tested prior to and after purifying, using trypan blue exclusion. The viability was 93%. Culture medium was William's E medium, hormone-free without dexamethasone and insulin, according to Witte et al. (12). To investigate the effect of T3, the hepatocytes were incubated for 6 days in three experimental setups: 1. invariant high hormone concentrations (INVARIANT; day 1–6: 10 μg/ml); 2. variant hormone concentrations according to physiological blood levels antepartum (VARIANT; day 1–6: 10, 9, 8, 7, 6, and 5 ng/ml); 3. control without any hormonal additive (CONTROL [CTRL]; day 1–6: 0 ng/ml). T3 (3,3″,5-Triiodo-L-Thyronine Sodium, T6397, Sigma-Aldrich Merck KGaA, Darmstadt, Deutschland) was added to the culture medium, which was changed every 24 h.

Cultured hepatocytes were daily morphologically evaluated by brightfield microscopy. Cells and supernatant a were collected on days 1, 4, and 6 and stored at −80°C until further analysis. Day 0 is defined as the day of isolation of the hepatocytes. Expression of GHR and IGF 1 mRNA was determined using a quantitative reverse transcriptase PCR. Total RNA was extracted using TRIzolTM Reagent (REF 15596018; Life Technologies Corporation, Carlsbad, USA), as previously published by Ehrhardt and Schmicke (13). The concentration and purity of extracted RNA were measured using a NanoDrop (NanoDrop 2000c, Thermo Fisher Scientific, Waltham, Massachusetts, USA) before transcription of RNA into complementary DNA (cDNA). PCR was performed using the Rotor-Gene Q (QUIAGEN GmbH, Hilden, Germany). Primers for GHR1A and IGF-1 were previously used and validated (12, 13).

## Results

**Figure 1:**
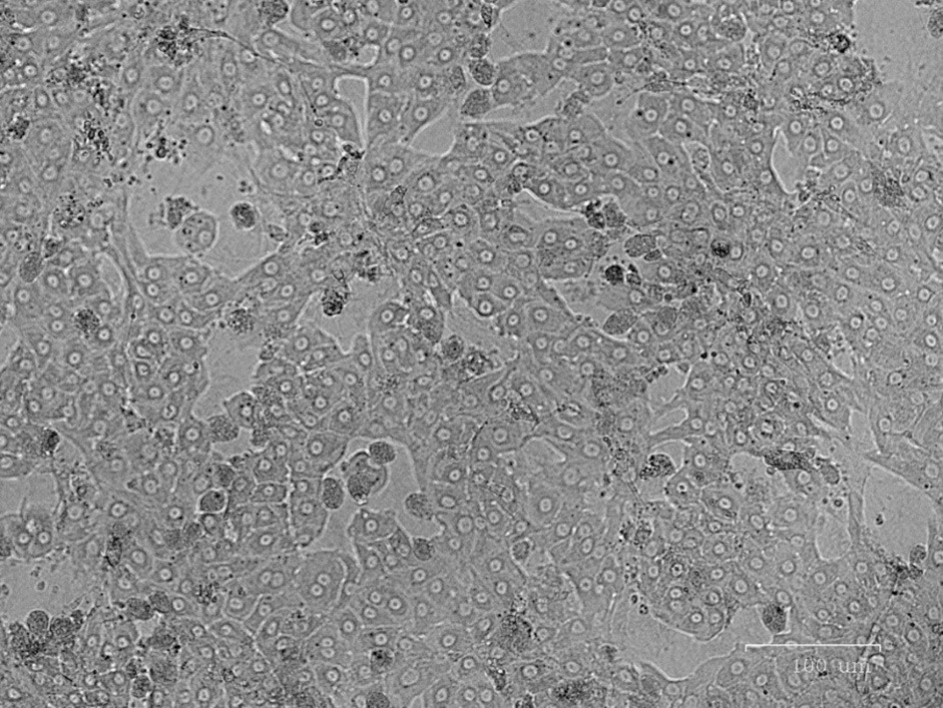
Primary bovine hepatocytes in hormone free control (CTRL) on day 4 of cell culture.

**Figure 2:**
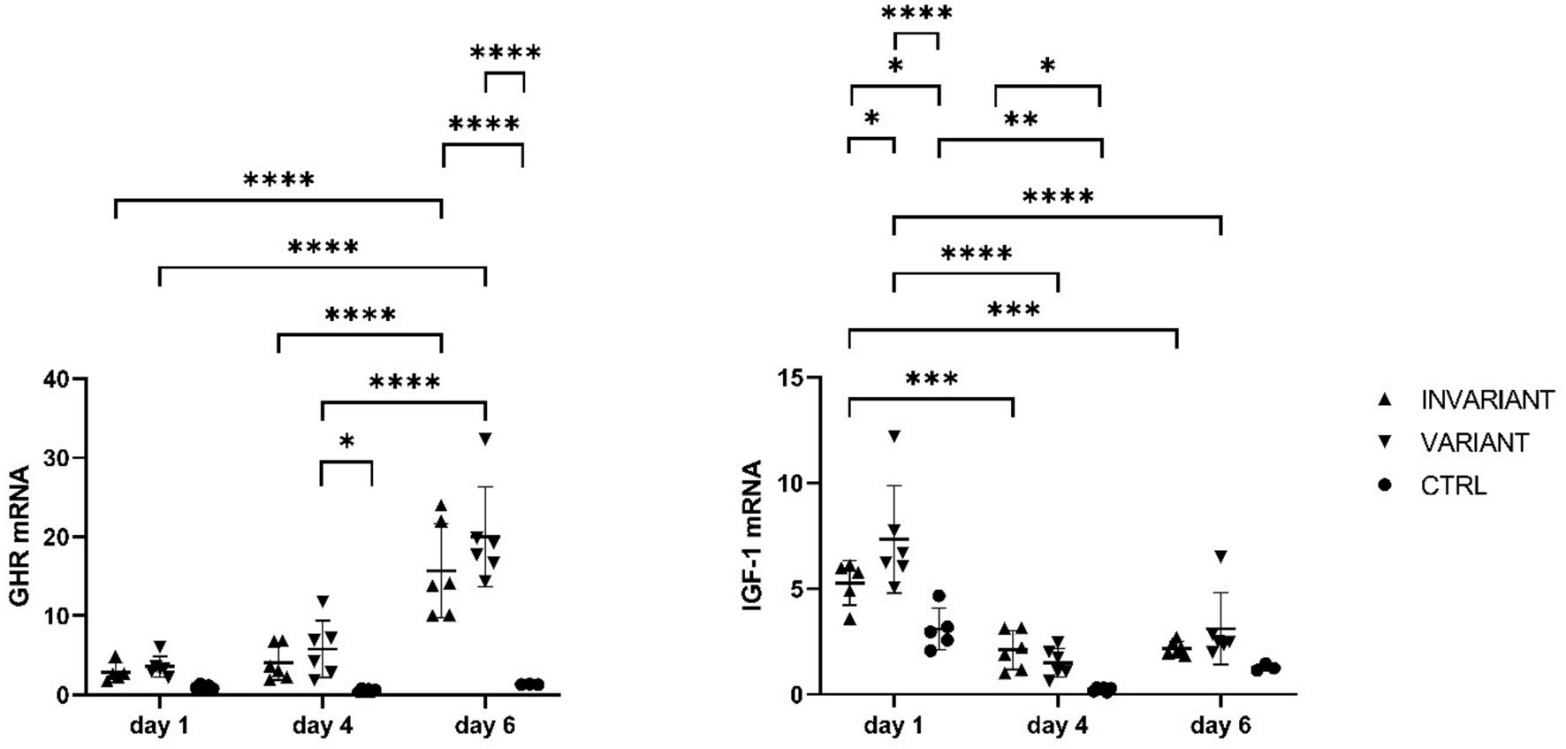
Influence of T3 on GHR and IGF-1 mRNA expression in primary bovine hepatocytes. Relative mRNA expression in adult primary bovine hepatocytes after culturing 1, 4, and 6 days with high (INVARIANT) or varied (VARIANT) concentrations of triiodthyronine and no hormone substitute (CTRL). *P < 0.05, **P < 0.01, ***P < 0.001, ****P < 0.0001.

In primary bovine hepatocytes (Figure 1) gathered from an adult dairy cow, T3 leads to an increase in GHR-mRNA expression, as well as IGF-1-mRNA expression, compared to the control (Figure 2). The changes in GHR expression after adding T3 were irrespective, if T3 was added in constant concentrations over 6 days (INVARIANT) or if T3 was added in decreasing concentrations (VARIANT) mirroring the decrease in T3 antepartum. Cell morphology of the hepatocytes did not change after T3 addition to the culture media (Figure 2).

## Discussion

We could confirm that there is an additive effect of T3 on the expression of GHR and IGF 1 mRNA in primary bovine hepatocytes, using a well-established cell culture model (12–14) with primary hepatocytes derived from an adult dairy cow. A pivotal question is how T3 can induce IGF-1 production in primary hepatocytes without growth hormone (GH) being added to the cell culture medium. An unaware substitution of GH to the cell culture by FBS can be ruled out because of CTRL that was treated equally while cultivation, afterward no FBS was added to the culture medium. Also, a cellular stress-induced alteration of mRNA expression can be ruled out because of CTRL. We hypothesize there is another unknown stimulus in the cell culture that mediates alterations of basal gene expression because similar effects occurred in Endriß et al., who also did not add GH to the medium (15). Over time IGF-1 mRNA expression increased and GHR expression decreased in T3-stimulated cells. This might be because GHR expression is not further stimulated due to the lack of GH in the medium. It also can be a specific effect of T3 on GHR and IGF-1 mRNA expression at nuclear thyroid hormone receptors to modulate transcriptional activities *via* thyroid hormone response elements in the regulatory region of target genes. The mechanism behind this should be deeply studied further. From that, *in vitro* data, it can be speculated that in cows with lower T3 less stimulatory effect on the GHR is present and this may further uncouple the somatotropic axis. This effect was previously indicated also in hypothyroid rats, which had a distinct lower binding capacity to GH in the liver (16). In chickens, the treatment with propylthiouracil leads to hypothyroidism (T4 and T3 low) and this leads to lower GHR and IGF-1 mRNA expression in the liver (17). It must be critically discussed that the lead exposure of the animal may influence the pituitary-thyroid hormone axis as was shown in men. Lead exposure alter TSH levels (18), but no hint was found that mechanisms at GHR or IGF-1 expression might be affected. As our results were in strong agreement with data from mice, humans, pig, and other species the effect of lead contamination might be insignificant. However, further studies are needed to reveal the impact and pathomechanism of T3 in the context of ketosis.

## Data Availability Statement

The raw data supporting the conclusions of this article will be made available by the authors, without undue reservation.

## Author Contributions

VS performed the experiments and wrote the manuscript. MS conducted the study, manuscript writing, and manuscript correction. All authors contributed to the article and approved the submitted version.

## Funding

This research received funding from the H. Wilhelm Schaumann Foundation.

## Conflict of Interest

The authors declare that the research was conducted in the absence of any commercial or financial relationships that could be construed as a potential conflict of interest.

## Publisher's Note

All claims expressed in this article are solely those of the authors and do not necessarily represent those of their affiliated organizations, or those of the publisher, the editors and the reviewers. Any product that may be evaluated in this article, or claim that may be made by its manufacturer, is not guaranteed or endorsed by the publisher.
